# Peripheral blood Th9 cells and eosinophil apoptosis in allergic asthma patients

**DOI:** 10.1186/2045-7022-4-S2-O11

**Published:** 2014-03-17

**Authors:** Deimante Hoppenot, Kestutis Malakauskas, Simona Lavinskiene, Ieva Bajoriuniene, Virginija Kalinauskaite, Raimundas Sakalauskas

**Affiliations:** 1 Lithuanian University of Health Sciences, Department of Pulmonology and Immunology, Kaunas, Lithuania

## Background

Th9 cells are novel identified subset of CD4+ T helper cells, which could contribute to airway inflammation in allergic asthma. The role of Th9 cells and their interaction with eosinophils is still not fully understood. The aim was to evaluate peripheral blood Th9 cells and eosinophil apoptosis in patients with allergic asthma.

## Material and Methods

18 patients with allergic asthma (AA) and 14 patients with allergic rhinitis (AR) were examined. The control group included 16 healthy subjects (HS). All AA and AR patients did not take inhaled corticosteroids at least for one month and/or histamine antagonists at least for one week. Peripheral blood eosinophils and CD4+ cells were isolated by high density gradient centrifugation and magnetic separation. Th9 cells and apoptotic eosinophils were estimated by flow cytometer. Serum interleukin-9 (IL-9) and interleukin-5 (IL-5) level was determined by ELISA.

## Results

Percentage of peripheral blood Th9 cells was increased in AA patients compared with AR patients and HS group (0.73 ± 0.32 % vs. 0.51 ± 0.10 %, 0.16 ± 0.08 %, p < 0.05, accordingly). The same tendency was observed for serum IL-9 level in all groups (75.6 ± 9.1 pg/ml vs. 50.5 ± 9.7 pg/ml, 37.8 ± 4.7 pg/ml, p < 0.05, accordingly). Percentage of peripheral blood apoptotic eosinophils was decreased in AA and AR patients groups compared with HS group (3.42 ± 1.29 %, 4.85 ± 0.97 % vs. 7.83 ± 1.40 %, p < 0.05, accordingly). Serum IL-9 level significantly correlated with percentage of Th9 cells (r = 0.64, p < 0.05) and negatively with percentage of apoptotic eosinophils in AA patients group (r = -0.58, p < 0.05). The negative correlation was found between apoptotic eosinophils count and IL-5 level in AA group (r = -0.76, p < 0.05).

## Conclusions

Airway inflammation in allergic asthma was accompanied by increased peripheral blood Th9 cell count and IL-9 level. Eosinophil apoptosis was inversely related to serum IL-9 level.

